# ZBP-MM: Zone-based producer mobility management protocol in named data networking for Internet of Things

**DOI:** 10.1371/journal.pone.0320654

**Published:** 2025-05-23

**Authors:** Shahzad Rizwan, Aatikah Rasool, Farhan Aadil, Salabat Khan, Zeshan Iqbal, Ghada Atteia

**Affiliations:** 1 Department of Computer Science, COMSATS University Islamabad, Islamabad, Pakistan; 2 Department of Computer Engineering, Sivas University of Science Technology, Sivas, Turkey; 3 Big Data Research Center, Jeju National University, Jeju, Republic of Korea; 4 Department of Information Technology, College of Computer and Information Sciences, Princess Nourah bint Abdulrahman University, Riyadh, Saudi Arabia; Virtual University of Pakistan, PAKISTAN

## Abstract

The Internet of Things (IoT) is a diverse technology that primarily utilizes TCP/IP protocols in IoT environments. However, it is often argued that the existing IP protocol stack is insufficient to support data sharing and storage capabilities for mobile IoT applications. Named Data Networking (NDN) has gained significant attention over time as a solution to the limitations of host-centric services in traditional IP networks. The key advantages of NDN include name-based routing and network caching, along with mobility support in wireless networks. The integration of NDN and IoT can address issues related to frequent node mobility, name-based routing, and storage constraints on resource-limited devices. IoT devices are frequently in mobility, creating routing challenges when transitioning from one domain to another. Interest packets remain unsatisfied until the producer reconnects to a new Point of Attachment (PoA) and updates its location. Despite these reconnections, challenges such as interest and data packet losses, increased latency, and suboptimal routing paths persist during communication. Consequently, managing producer mobility remains difficult during the frequent movement of nodes across different IoT domains. This paper proposes a novel Zone-Based Producer Mobility Management (ZBP-MM) protocol for NDN-based IoT environments. The proposed technique is evaluated and compared against the OPMSS scheme using the ndnSIM simulator. Results demonstrated that the proposed scheme achieves better hop count and cache hit ratio, with minimized interest satisfaction delay and handover delay, compared to the OPMSS technique during producer mobility scenarios in intra- and inter-zones.

## Introduction

**Fig 1:**
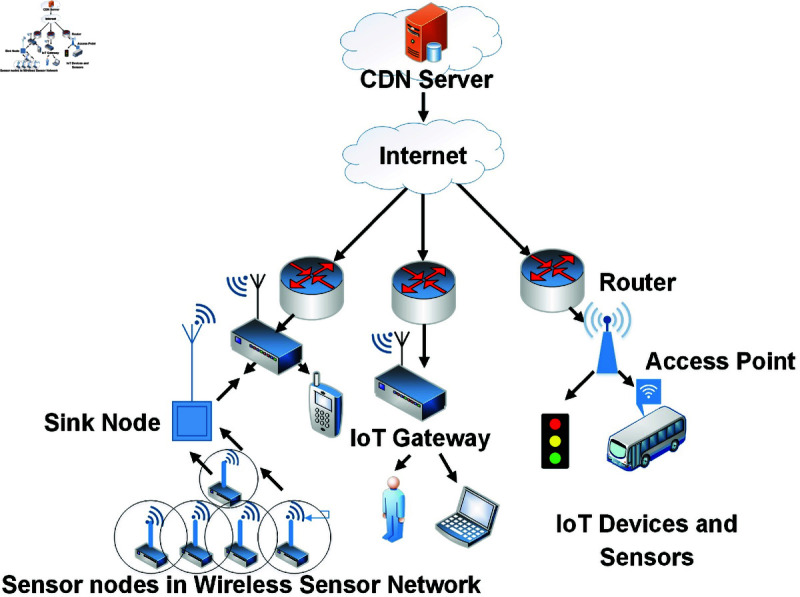
NDN based Internet of Things environment.

Named data networking (NDN) [1] is a widely used and popular Future Internet Architecture of ICN [2] in the field of the computer network to replace the shortcomings of traditional IP-based network architecture [3]. In NDN architecture, the contents are name-based instead of location-based. The NDN consists of routers called NDN or Content Routers, which can cache the data based on the cache replacement policy in the network. A consumer can find the required content from the closest router’s cache along the path as NDN mainly uses “Caching Everything Everywhere (CEE)” [4]. Otherwise, a request is forwarded to the original content producer. On returning, the data packet is cached on-path routers, as discussed earlier. The in-network caching support helps in reducing the redundant network traffic for requesting the same content from the producer. The Internet of Things (IoT) [5] ecosystem is considered a central part of the next-generation network for promoting smart-based devices. These IoT devices are software-enabled which can communicate through the network and exchange data. However, smart devices have many limitations, such as low memory, computational power, and low energy devices [6]. These constraints may create a communication problem on such devices. On the other hand, the Broadband IoT, LTE Mobile (LTE-M), and Narrowband IoT (NB-IoT) are part of the cellular or mobile IoT defined by Ericsson [7]. Mobile IoT devices include drones, vehicles, wearable biosensors, trackers, and augmented and virtual reality devices. These devices generally have long battery life, large communication range, and high data rates, making them highly suitable for Wide Area Network (WAN) use cases. The mobile IoT devices mainly use LTE- M or NB-IoT technologies that co-exist with 2G/3G/4G [6]. Traditional IP-based network architecture has faced many challenges in IoT networks, especially in providing mobility management and security support. The NDN can cope with such IoT network challenges at the network layer [8]. The NDN architecture is a comprehensive solution for scalable IoT due to its named-based resolution and routing, intermediate caching, and producer mobility support [9]. However, the NDN solves the host-centric IP-based communication problem through a data-centric approach. Thus, many researchers have investigated that NDN can be a more flexible and robust solution for IoT networks for providing content-based routing and in-network caching support [10,11]. Moreover, as IoT devices are frequently mobile, it is essential to keep uninterruptible network connectivity. During network communication, whenever an IoT device moves to another location, the interest packets destined for the producer are unsatisfied until it re-establishes a connection with another Point of Attachment (PoA) and re-broadcasts its new location in the whole network [12]. Fig 1 illustrates the IoT devices and sensors in NDN environment.

**Fig 2:**
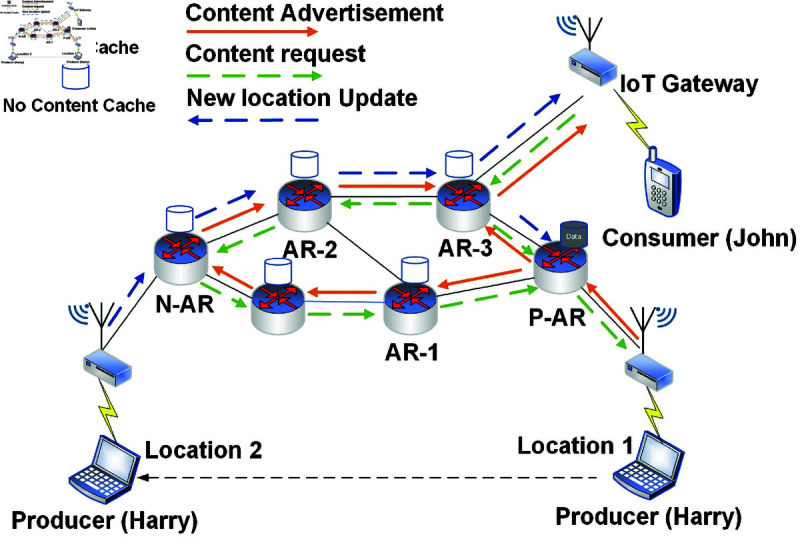
Producer mobility concept in NDN-based Internet of Things environment.

Mobility support is crucial for mobile IoT devices such as smartphones and wearable biosensors [13], where data connectivity is critical. Concerning publisher mobility, FIB table entries need to be updated, and name prefixes for the named data must be re-advertised via the routing protocol over AR. However, re-advertisement requires a re-registration process when the producer changes its locations, but such a process experiences a huge delay overhead in relaying messages to the AR. Fig 2 illustrates the basic concept of producer mobility in the network. Consider the scenario, where a consumer “John” requests specific data by sending an interest packet in the network.

At first, the content router searches for the required data in its cache. In case of a match, the router replies to the consumer. Otherwise, the interest messages are propagated in the whole network till received by the producer “Harry”. The producer “Harry” replied with the data packet through the Previous Access Router (P-AR) to Access Router 1 (AR1) and subsequently from AR2 to AR3 till the consumer “John”. During the communication, if the producer moves out of the network, it results in interest packet satisfaction failure at P-AR. i.e. Harry’s disconnection from P-AR. When the producer connects with the Next Access Router (N-AR), it broadcasts its new location to update the new routing path in the network. On the other hand, the consumer resends the unsatisfied packets, which will be only satisfied after establishing a new updated routing path. i.e. from N-AR to AR2 and till the consumer. In the case of consumer mobility, depending upon the handover process, a node reissues new or unsatisfied interest messages from its current or new location. i.e., before and post-handover. After the handover process, the required data messages will be delivered at the consumer’s location with higher end-to-end delay and lower throughput due to the flooding process in NDN.

The proposed ZBP-MM technique has been designed to meet the following objectives:

- To propose a novel Zone-based Producer Mobility Management Protocol (ZBP-MM) for NDN-based IoT environment to help Zone Access Routers manage the producer mobility in intra and inter-zones.
- To minimize the interest satisfaction delay in a producer mobility scenario.
- To suppress the interest broadcast in sparse, less dense, dense, and very dense topological scenarios within the zones.
- To minimize the cache size overhead and reduce the packet loss in the network during producer handover.

The paper is organized into the following sections. Section Related work describes the related work. Section Related work presents the problem statement. Section Proposed ZBP-MM protocol discusses details about the proposed work. Section Performance evaluation presents the performance evaluation. Finally, Section Conclusion describes the conclusion and future directions.

## Related work

**Fig 3:**
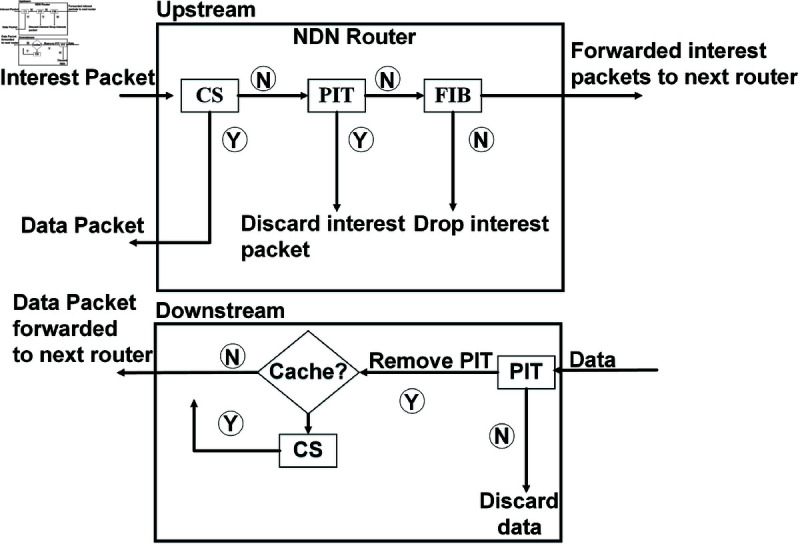
Interest and data forwarding in NDN routers.

To achieve the named-based routing and caching, NDN uses three types of data structures for NDN traffic management that include Content Store (CS), Pending Interest Table (PIT), and Forwarding Information based (FIB) [14]. Any consumer in the NDN broadcasts the interest messages for the required content that passes through many intermediate content routers. Upon receiving, any content router looks for the content in its CS, and if found, the router replies to the consumer. Otherwise, check for PIT entries for the same content. If a PIT entry already exists, then the router drops the interest. Otherwise, the router adds a new PIT entry and looks for a FIB entry. If the FIB entry already exists, then it drops the interest packet. Otherwise, adds a new FIB entry and forwards the interest to the next-hop router. On downstream, the intermediate router caches the content in their CS along with the delivery path and forwards it to the consumer according to the corresponding PIT entries. Fig 3. shows the standard NDN interest and data forwarding process.

Based on the hierarchical namespace prefix naming, a path update problem occurs when any producer moves and connects to a new location. During the handoff process, the interest packets remained unsatisfied until the producer re-connected to a new PoA. After the handoff, the new router broadcasts the producer’s new location to all the nodes in the network for path update. But such path update results in high overhead [15]. During the handoff process, a consumer may face a long delay in data retrieval as a frequent change in the location of nodes causes PIT, FIB, and CS entries to change at an intermediate node in the network. Many authors discussed solutions regarding producer mobility. Some of the techniques focused on path updates after the handoff, while others proposed reducing the number of interest and data packets lost during the handoff process. To manage challenging issues such as reverse path partitioning, message broadcast storms, and sporadic connectivity due to frequent topological changes in NDN-based VANET. Zafar *et al*. proposed a DRLSF protocol for NDN-based VANETs [16]. The protocol uses a contention-based priority forwarding mechanism, selecting high-priority vehicles based on link quality, link lifetime, direction, and distance to the previous forwarder. The proposed scheme introduces a distinct zone-based forwarding that divides the road into different zones based on angular information, expanding the interest message forwarding process to locate the producer. It also features a forwarding backing set (FBS) of a few nodes in the neighbourhood of the potential interest message forwarder, creating PIT entries to forward the data message in exceptional scenarios where the original intermediate forwarder leaves the communication area, reducing the likelihood of data message drop in the network and avoiding unnecessary interest message retransmissions by consumers.

By employing a neural network model to precisely anticipate producer locations and route computations, Rui *et al*. present a Double-Lead content search strategy that is based on neighbour, proxy, and location prediction [17]. In order to minimize network overhead and delay, the Double-Lead content search method (DLNDN) combines producer passive notification with consumer active search. For prediction accuracy, a deep learning method is also used. In their plan, they added two more tables to the already-existing data structures, four more kinds of interest packets, and more columns to the FIB database. Fayyaz *et al*. proposed a hybrid mobility management scheme for named data mobile ad-hoc networks (SHM-NDN) to address producer and consumer mobility management challenges in NDN-based MANETS [18]. Their scheme restructures wireless FIB and adds five new packet types to facilitate effective mobility management. Producer mobility management is separated into two stages: network partitioning owing to unstable conditions and stable wireless channel management. The introduction of a path recovery technique addresses network partitioning problems. A PIT-only forwarding technique is also used by the SHM-NDN to facilitate customer mobility in MANETs. The development of five new packet types, a path recovery technique, an effective controlled packet forwarding system, and a PIT-only forwarding mechanism to address network partitioning difficulties are the primary contributions. Kar *et al*. proposed a Neighborhood Registration Scheme (NRS) for producer mobility in NDN-based healthcare monitoring system [19]. Their proposed scheme registers the mobile producer with many neighboring Access Points (APs) in addition to the closest AP. The paper proposes a handover scheme and algorithms to shorten latency caused by handover and eliminate delays caused by mapping system updates. Selection choice update evaluates the distance between mobile producers and nearby APs, while hand-off detection helps detect hand-offs and reduces latency. Neighbourhood update allows mobile producers to register themselves into multiple APs, allowing them to move across multiple cells without worrying about latency. Hernandez *et al*. introduce a Named Data Network (NDN)-based solution for consumer mobility [20]. It involves a mobility manager that tracks and predicts trajectories, allowing infrastructure to adapt to new routes and manage customer movement more effectively while also providing higher-quality services. The main objective of this work is to design and implement a network entity for network-assisted content distribution that tracks the movement of mobile customers across time. Additionally, it suggests a way to guarantee smooth handover by facilitating consumer mobility in an NDN-based architecture via a remote mobility management entity. Validated by simulation results, the NDN-based solution improves service quality in highly mobile situations based on real and fake vehicle mobility and connectivity patterns.

Prates *et al*. [21] proposed a GeoZone scheme that forwards the interest packets to the zone referenced by the Global Positioning System (GPS) coordinates of the content. GeoZone scheme assumes the position of the requested content ahead of the consumer travel direction and coordinates in the dissemination zone. The proposed GeoZone scheme restricts the flooding problem to a small network zone. In the non-proactive GeoZone approach, only nodes in the dissemination zone can store and forward the data packets but only if the producer node is present in the dissemination zone. Otherwise, the forwarding nodes do not have name prefix information where the producer for the desired content, when the producer moves away from the dissemination zone. Thus, non-proactive-GeoZone suffers from high cache misses due to the away movement of the producer(s) from the zone. On the other hand, in the proactive-GeoZone approach, the nodes forward the content proactively thereby storing data packets at the Content Store (CS) of each forwarding node in the zone. Thus, non-proactive GeoZone suffers from high cache misses, interest and delay overhead. On the other hand, proactive-GeoZone provides an increased cache hit ratio and probability of finding the requested name prefix in the network but increases cache replication at each forwarding node between source and destination. For deployments in large-scale intra-domain networks, the CRoS-NDN extension to a multi-controller scenario can be conceptualized as follows. The named data location storage task can be shared by several controllers, and each controller is aware of a subset of routers referred to as a zone [22] as well as the paths connecting the routers in its own zone. To determine the next zone hop to any other zone in the network, each controller needs to have a topological view of the nearby zones. Using the hash of the controller ID as the DHT node identifier and the hash of the specified data prefix as the DHT key, the controllers can be arranged in a distributed hash table (DHT). The router ID for the prefix is found in the DHT value. An index rule based on DHT node ID governs key distribution in DHT nodes. Rao Gao *et al*. [23] proposed the locator-based producer mobility scheme by extending the original NDN architecture packet structure and interest packets caching at the AR. During the producer handoff event, the producer sends a mobility packet to P-AR. The P-AR stops delivering the interest packets until the producer connects with the N-AR. The N-AR adds FIB entries for the producer while the P-AR updates its FIB also and sends cached packets to the producer’s new PoA via N-AR. Thus, the producer starts sending data packets to the consumer’s new PoA. The proposed technique reduces the interest packet loss and delay during the handover situation. However, it does not consider an optimal path between producer and consumer after handoff. Due to additional fields, the routing table scalability issues arise in large network scenarios. This technique is not scalable. Tang *et al*. [24] proposed a control and data plane separation approach in which the network consists of Rendezvous Domains (RDs), Resource Handler (RH), Rendezvous Point (RP) and Home Repository (HoR). During intra-domain producer movement, the RH allocates RP for interest and data forwarding towards the new location thus, avoid the global information update. On the other hand, during the inter-RDs producer movement, the RH notifies the RP and HoR regarding local and global information updates. The proposed scheme reduces the packet delivery cost by avoiding Location Update (LU) packets to HoR for the producer’s new location. However, the proposed technique causes high latency during communication with HoR for global producer information sharing. Do and Kim [25] proposed an optimal handover scheme for the mobile producers to predict and establish an optimal path before the initiation of the handover process. In the proposed technique, the producer can connect with the next-router after getting strong radio signals. The current router sends the producer’s previous hop and next-hop information back to the consumer. The consumer updates the route by deleting the older routing path through the intermediate router. Thus during the producer handover, the new router forwards already cached interest packets towards the new update path. The proposed mechanism reduces the interest packet loss but incurs signalling overhead in a large-scale network deployment. Zheng *et al*. [26] proposed a technique with a multi-level anchor router path to relay interest packets to the consumer. Any mobile producer informs the previous router about the expected handover after detecting signals from the next router. The current router informs the crossing anchor node that establishes a path with the next router before forwarding interests on routing paths. However, upon completion of the handover process, the router deletes the previous router path and allows the interest packets to follow the new updated path. The proposed technique reduces the handover latency and improves response time by establishing the forwarding path in advance. However, forwarding the interest to multi-points causes unnecessary bandwidth utilization and redundant transmission.

Ravindran and Wang [27] proposed a distributed anchor-based architecture and decentralized micro-level resolution system. The domain system consists of local and global anchor routers for handling name-based forwarding and on-demand producer mobility within the cluster-based domain hierarchy. The proposed technique establishes an efficient interest forwarding mechanism by using namespace mapping to the next anchor in the corresponding domain and data packets using overlay-based forwarding during the handoff process. However, the proposed technique showed high signalling cost and bandwidth consumption due to pre and post-handoff of the producer. This scheme is not efficient. Ge *et al*. [28] proposed an SDN controller-based solution to update FIB with an optimized path based on producer mobility patterns and a new network state. The proposed technique leverages the SDN controller to detect the current location of the producer. i.e. from the previous to the new router. SDN controller also defines a new FIB update with an optimal and shortest path of the corresponding routers. The proposed solution minimizes request-response latency and reduces control overhead. However, additional devices are needed to maintain address mapping information. Moreover, the distance between the controller and routers may cause high latency overhead. This scheme is not cost-effective hardware-wise. Rui *et al*. [29] proposed the proactive-based caching scheme, in which the producer sends a packet to the router before the handover decision. The proposed solution consists of a proactive data caching method at the current router and an interest messages pull strategy towards the next router to manage efficient packet delivery. The proposed schemes reduce handover delays due to proactive caching and pull approaches. However, keeping contents in the cache (i.e. without being replaced or modified) for a longer time without an efficient caching policy is an issue. Also, the scheme suffers in terms of handover delay. This scheme is not scalable with proactive caching when the network size increases. Yu *et al*. [30] proposed KITE, a producer mobility tracing approach implemented through the Rendezvous server (RV). RV performs name-based mapping and hop-by-hop breadcrumb path establishment approach with producer and consumer. The producer issues Trace Interest (TI) with a “Trace” tag to the RV. The RV authenticates the TI and broadcasts Trace Data (TD) for the intermediate routers to update FIB entries according to the producer prefix. KITE makes the producer’s new location transparent through anchor routers. RV uses a distributed manner approach to improve the triangular path issues and minimizes handoff delays. However, when the number of nodes increases, thus maintaining the trace path creates significant network overhead. Moreover, KITE experienced higher signalling cost overhead due to higher producer stability at a similar location. Jordan, *et al*. [31] proposed MAP-Me, a distributed anchor-less approach. In the MAP-Me technique, the producer sends a request to update FIB entries after the handoff process. MAP-Me updates the minimum nodes at the edge, activated by the producer node during its location change. The proposed technique helps to reduce the handoff latency and signalling overhead. However, the path stretching value is below average. Moreover, as MAP-Me architecture is regarded as micro producer mobility, thus the producer mobility is handled within the same Autonomous Systems (AS).

Hussaini *et al*. [32] proposed an optimal broadcasting strategy that introduces a Mobility Interest (MI) packet that is broadcasted to the Anchor Router (AR) for forwarding hint information during producer mobility. After the successful handoff, the AR broadcasts the MI packet to the intermediate routers to update their FIB entries according to the new location of the producer. The proposed technique reduces handoff latency, but it is only applicable in Wireless networks. However, the proposed technique offered high bandwidth consumption due to excessive broadcasting and unsatisfied interest delay. Ali and Lim [33] proposed an anchor-less location prediction technique that notifies the future expected location and handoff probability of the mobile producer to the nearest Access Point (AP) with the highest Received Signal Strength (RSS). All the APs are within a known location in the network. AP, to which it was initially attached, redirects interest packets to the predicted AP after the completion of the handover process. The proposed technique reduces handover latency and end-to-end round-trip time with minimum network overhead. However, the prediction accuracy is 50% which may result in prediction failure. Moreover, broadcasting the packet for location updates is excessive in large networks. Farahat and Hassanein [34] proposed a proactive caching scheme that caches the content in advance by predicting the producer handover event. The proposed technique also anticipates the consumer’s future location and caches the data in advance at the routers nearest to the consumer. Whenever the handover is detected, the potential interest packets are dropped and cached the requested data in advance. Reserved space is created in CS to cache mobile request data. The proposed technique reduces data delivery delay and increases response time. However, the proposed scheme fails to support longer handoffs due to limited cache size and failed handover prediction. Araújo *et al*. [35], proposed the SCaN-Mob technique to minimize the data unavailability during the producer mobility by alleviating content caching on selected nodes. SCaN-Mob selects the edge and access nodes in the vicinity for storing the content on the basis of a round-robin fashion upon receiving the request. The proposed technique improves the interest satisfaction rate, delivery delay and cache hit rato noteably. However, memory wastage is observed due to many replicas on the network nodes. Moreover, no routing path update strategy is presented when any mobile producer enters the network and connects to another PoA. Maroua *et al*. [36] proposed the AFIRM technique to update the forwarding information during the producer mobility. The FIB table is modified to store the outgoing interface to update the existing FIB entry with the least-hop distance and data routes from a different interface. The proposed technique uses the keep-alive movement detection method using the ping method to handle the mobility of the connected sensors. Moreover, it reduces packet loss due to the caching strategy while the consumers are satisfied with cached data over the request message path. However, the packet loss is higher due to the flooding approach because AFIRM does not use the flooding method to update the forwarding information in all nodes.

Hussaini *et al*. [37] proposed a producer mobility management scheme to update the routing paths using Mobility Interest (MI) packets. Using MI packets, the intermediate routers are updated. Thus, the consumer resends the unsatisfied interest packets to the anchor router, while the current anchor router sends the interest packets to the producer’s new location after handoff. The proposed scheme minimizes the packet delivery, latency and signalling costs with increased hop count distance during handoff situations. However, this scheme suffers in terms of performance parameters during the frequent mobility and lowest pause times. Hussaini *et al*. [38] proposed an optimal producer mobility support scheme (OPMSS) using the hierarchical namespace with movement prefix names and the mobility-based status flag. The Content Router (CR) updates or adds a new prefix name and mobility flag regarding producer mobility by broadcasting MI packets under the domain. The proposed scheme shows lower handoff latency, signalling cost and higher packet delivery costs for intra-domain producer mobility by choosing the best or optimal path between the consumer and the producer. However, the proposed scheme offered additional time in the FIB table update and optimal path selection when the hop distance between the consumer and the producer increases in the inter-domain. Gohar *et al*. [39] proposed a cluster-based device mobility management scheme for producer mobility. The current AR sends the producer’s new location information to the new AR, which sends the attachment information to the cluster head. The cluster head caches the content on behalf of the producer and is responsible for exchanging periodic updates of the connected nodes in the network. The proposed scheme maximizes the interest packet satisfaction ratio through the cache by diverting the interest packets to the AR. However, the overhead of cluster head selection algorithms using different approaches is difficult, especially in wireless networks. Swaroopa and Chilukuri [40] proposed a CCN scheme that uses a hierarchical naming scheme with prefix matching. The edge router uses caching replacement policy based on calculating the popularity of the data objects in terms of trendiness and frequency values at each router. The edge router checks previously cached or not cached data object based on the trendiness or popularity of the data object. However, the proposed scheme does not consider unpopular data and is affected by the reduced cache size due to redundant data. Oussama *et al*. [41] proposed label-based mobility support for the producer during the handovers. The FIB entry contains an additional field, the label generated through a hash function. The routing of the interest packet is performed througsignalling identification rather than on the original name by using relevant label FIB entry lookup to reach the mobile producer. The proposed scheme provides a location-free based naming scheme for handling producer mobility. However, this scheme offers hash value calculation overhead as the number of interfaces increases. Furthermore, there is no rule defined for a location-based hierarchical namespace.

## Problem statement

Named Data Networking (NDN) is an emergent and promising realization of the Information-Centric Networking (ICN) architecture for provisioning host mobility (i.e., both producer and consumer) in a location-independent manner. Regarding consumer mobility, unsatisfied interest packets become stale during movement and must be re-sent from the new location [25,37]. Although NDN inherently supports consumer mobility [1], producer mobility poses significant communication challenges during changes in network location or domain and connections to new Points of Attachment (PoA) during handover situations. Additionally, frequent producer mobility disrupts pre-established name resolution, data routing paths, and communication links, increasing the likelihood of handover latency, signaling costs, packet loss, and unnecessary dissemination of interest messages [21,30–34,37]. Mobility support is a critical requirement for NDN-based IoT networks due to the heterogeneous communication among sensor devices, network technologies, and service applications. A standardized NDN-based IoT architecture requires an access point attached to a content router to facilitate access to relevant content or services. In addition, the content router facilitates data sharing with interoperability between consumers and producers in a real-time session environment.

Regarding producer mobility, there are four major approaches: Anchor-based approach [26,27], In this approach, NDN routers act as a chain of anchor nodes. These anchors are responsible for updating the producer’s path in the network during mobility. The primary issue arises when an anchor node fails, leading to communication path breaks and potential network breakdowns. Anchor-less approach In this method, the producer informs the network of its location without relying on specific routers to manage mobility. This approach avoids packet broadcasting after the handover process and is generally more efficient. Optimal and broadcast-based strategies [32,37,38]. [20,21]: Here, all intermediate router FIB entries are updated after the handover process. However, this can create network overhead, particularly if the producer’s movement is frequent and continuous. Mapping-based approaches, These involve an SDN controller [28], rendezvous servers [24,30], zone-based [22], or cluster-based strategies [39]. These servers are responsible for mapping the producer’s route in the network. However, communication over longer distances between the consumer and the server may result in high handover delays and latency. Despite these solutions, challenges such as interest satisfaction delay, handover signaling cost, and excessive hop counts during mobility remain significant issues for the NDN architecture.

Our proposed solution focuses on the above-mentioned problems to support producer mobility by organizing nodes into zones with varying device densities. Initially, no nodes act as content producers until a node publishes content. When a node sends an interest request, the zone router checks its Forwarding Information Base (FIB) to determine if the request has been processed. If not, the request is forwarded to a border router and then to the CDN server, which supplies the requested content. The receiving node caches this data locally and updates its zone router, which propagates the information across other zones. This node is then designated a producer. A round-robin approach manages the cache due to its limited size. The ZBP-MM protocol improves upon OPMSS by reducing delays through efficient zone-based request routing and dynamic content sharing. Zone routers update one another when content information changes, enabling quicker responses. For multiple producers, requests prioritize proximity and responsiveness, defaulting to the CDN server if no producers reply.

## Proposed ZBP-MM protocol

### Proposed technique

**Fig 4:**
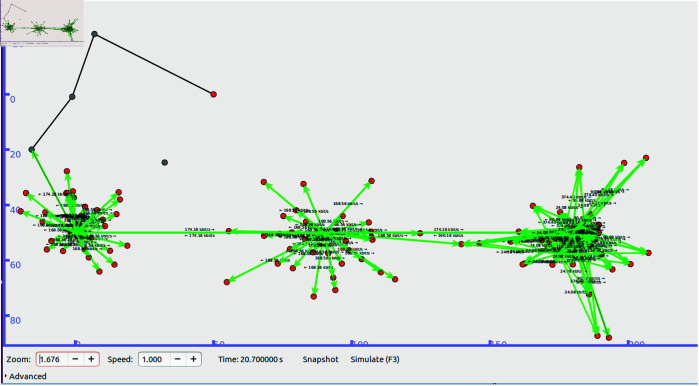
Proposed ZBP-MM protocol.

We have implemented the network comprised of 3 zones as shown in Fig 4.

Each zone contains varying densities of mobile devices. Initially, no node acts as a producer until a node publishes content within the zone. A node sends an interest request packet to its designated zone router. Upon receiving the request, the zone router checks its FIB entry. If a matching entry exists, it implies the interest has already been forwarded. Otherwise, the zone router forwards the request to the border router, which subsequently sends the request to the CDN server. The CDN server responds by sending the requested data back to the consumer node. Upon receiving the requested data, the respective node stores it in its local cache and shares the content information with the intra-zone router. Later, it disseminates this information to all other zone routers in inter-zones. Consequently, this node becomes a producer node, having published content within the network. We employed the round-robin technique to manage the cache, given that the cache size is considered limited.

In the OPMSS technique, content routers flood interest packets across the network until the content is found in the CS of an intermediate router or a potential content producer in the network. In cases of producer mobility within an intra-domain or inter-domain, the producer’s content prefix location is updated using mobility interest packets after the handover process is completed. However, this results in consumers being informed with significant delays, even after the intermediate routers receive mobility updates.

**Fig 5:**
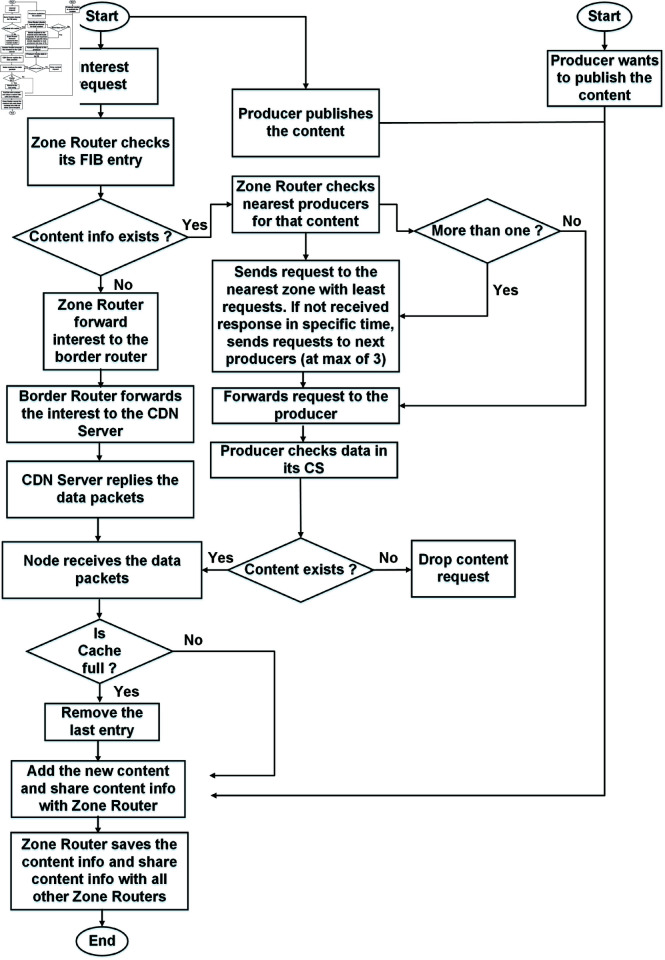
Flowchart of the interest and data packets transmission in the ZBP-MM network.

In contrast, the proposed ZBP-MM protocol reduces both interest satisfaction delay and end-to-end delay significantly. If no content is found or published at any zone router within the network, the content is fetched from the CDN server, as previously described. Additionally, the zone routers share content information with other zone routers whenever new or updated content information becomes available. Fig 5 presents the flowchart of interest and data packet transmission, as well as producer mobility in the ZBP-MM network.

If the content is available in the intra-domain or inter-domain, the previous zone router and the next zone routers exchange mobility update packets, ensuring a well-coordinated, predictive handover of the mobile producer across domains. In the OPMSS technique, when another consumer requests the same content from the domain router, the router broadcasts the interest request to the producer-connected router due to a lack of knowledge about the specific content source. In our algorithm, the node always sends the interest request to its zone router. The zone router determines where to forward the request based on FIB entries. Upon receiving the content request, the producer replies by sending data packets to the requesting node. Similarly, the receiving node stores the data in its local cache and shares the content information with its zone router. If more than one producer’s FIB entry is found at the zone router for the same content request, the zone router selects the best producer among the three nearest options based on request frequency. If the first producer fails to respond within a specified time, the zone router selects the next nearest producer. If the second producer also fails to respond, the zone router then attempts to fetch the content from the third nearest producer in the zone. If none of the producers respond, the content request is ultimately forwarded to the CDN server. In our technique, the producer is required to publish its content within the zone before any consumer requests it. Additionally, the zone router shares the new content prefix information with other zone routers, ensuring efficient dissemination of content information across the network.

### Topological scenarios

We have implemented sparse, less dense, dense, and very dense topological scenarios in intra- and inter-zones, with varying producer mobility cases as described in the next section. In a sparse topology, the density of both consumers and producers is 20%, resulting in low connectivity between them. In less dense and dense topologies, the density of consumers and producers varies from 30% to 40%, providing medium connectivity. In a very dense topology, the density of both consumers and producers is set to 50%, resulting in high connectivity.

### Producer mobility

**Fig 6:**
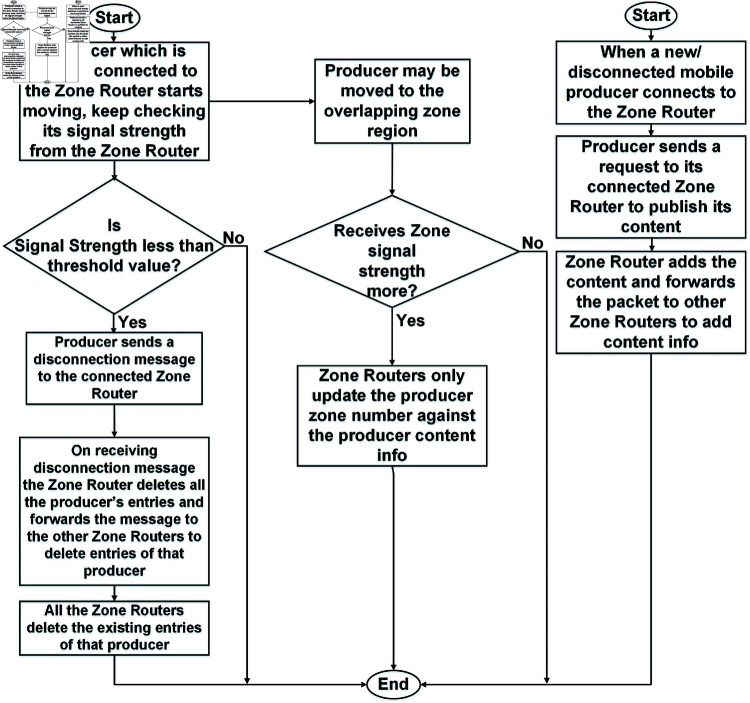
Flowchart of the producer mobility in the ZBP-MM network.

As discussed earlier, a producer node can be a mobile node within a zone. The producer can roam within the same zone or across adjacent zones. In our technique, a producer node continuously monitors its signal strength with the connected zone router. If the received signal strength consistently falls below a specified threshold, the producer sends a disconnection message to its currently connected zone router, informing it of the disconnection from the current zone network. Fig 6 illustrates the flowchart of producer mobility in the ZBP-MM network.

On receiving this packet, the zone router soft deletes the FIB entries of that producer and sends a packet to other zone routers to delete the respective producer’s entries. If the producer does not re-connect in a specific time limit, the zone router permanently deletes its FIB entries. Later on, if a producer re-connects at any zone router, then a producer node publishes the content with the connected zone router. Also, the zone router adds the producer’s entry information and shares it with the other zone routers. In another scenario, when a producer moves to another zone, it sends a Mobility Update (MU) packet to the new zone router. The new router updates the zone number against the producer’s record in its FIB. The zone router also shares the information with other routers the producer’s new location update in the FIB tables.

### Producer mobility scenarios

The following are the case scenarios handled by our proposed ZBP-MM protocol.

#### Scenario 1.

**Fig 7:**
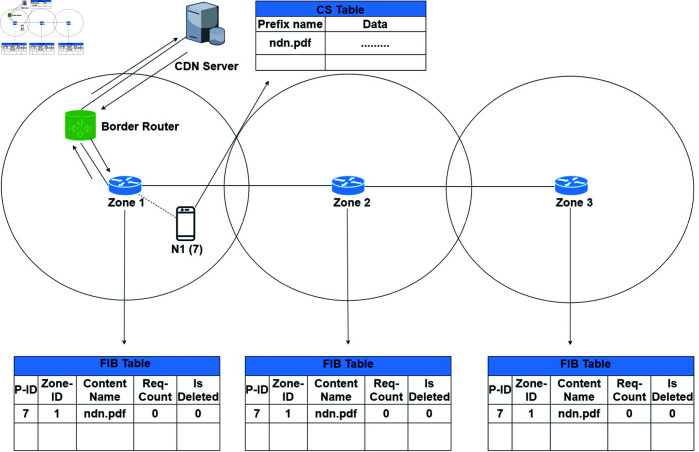
A node requests a content that is not available in the network.

A node N1 requests an interest packet like “ndn.pdf” to its zone router Z1. The zone router checks its FIB entry either the content is available in the network or not. In case of no content source information, it redirects the interest request to the border router. The border router sends the request to the CDN server. Now CDN server response backs the content “ndn.pdf” to the respective node N1. On receiving the content, the node saves the data in its CS and informs other zone routers to add content information in their FIBs. i.e. Z1 adds the content entry in its FIB, the incoming interface, the producer zone number, and the content name. Afterwards, it shares the recent content information with other zone routers, i.e. Z2 and Z3. Thus, Z2 and Z3 also have the producer content information as shown in Fig 7. Such a node now becomes a producer for nearby consumers to be satisfied with the content in the future.

**Fig 8:**
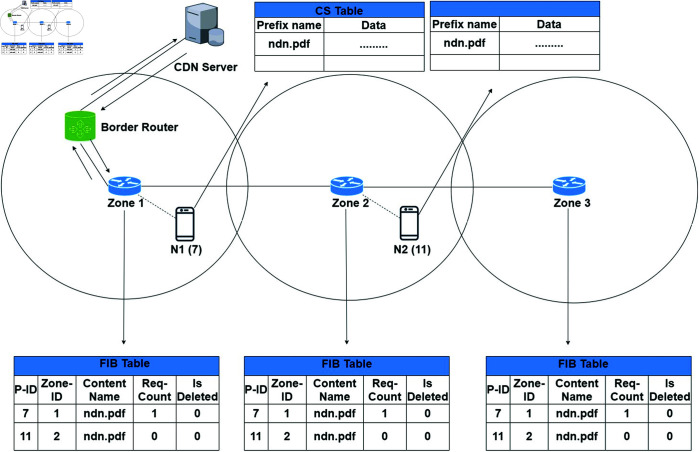
A node requests content that is available in the network.

Similarly, if any other node "N2" requests the same content “ndn.pdf” from the zone router Z2. Then the Z2 checks the content entry in its FIB and finds the requested content. The zone router redirects the request to the Z1 and afterwards to "N1". On receiving the content request, the producer N1 sends the data packets to the "N2" in the Z2. The receiving node "N2" performs the same steps as discussed above for the node "N1" as shown in Fig 8. Now the zone routers Z1, Z2 and Z3 all have the same content source information of two different producers. Thus, our proposed ZBP-MM technique increases content availability in the zone. Moreover, the ZBP-MM technique reduces the network disconnection and zone load balancing problem significantly. In other words, ZBP-MM reduces content fetching load on the zone routers as just content information reference is stored instead of actual content. Similarly, if any other node from either zone requests for the same content afterwards, then such a request will be satisfied in the future.

#### Scenario 2.

**Fig 9:**
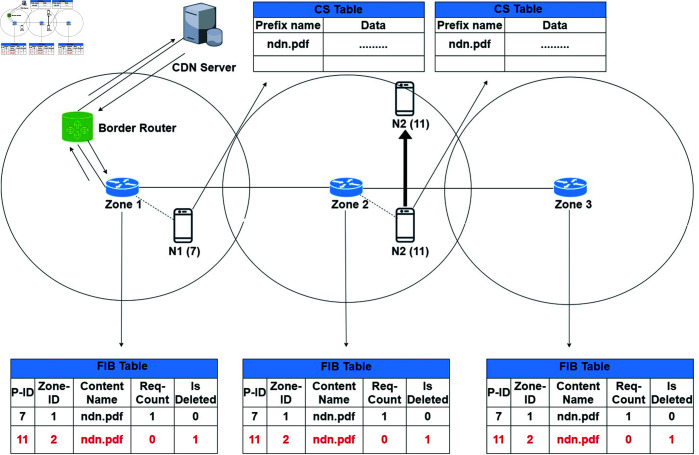
Producer node is disconnected from the network.

In this scenario, suppose a producer node "N2" moves in such a pattern that it passes the specific signal threshold limit and disconnects the Z2 network as shown in Fig 9. The producer then sends a disconnection message to its current zone 2 router. The Zone 2 router sets the IsDelete flag to 1 and shares the updated IsDelete flag information with other zone routers. On receiving the disconnection message, all the zone routers delete their entries of such producer nodes. But after a specific time interval, the router resets its soft delete entry to zero once more if "N2" reconnects with the Z2 network. Otherwise, the router deletes the IsDelete flag entry permanently. Formerly, after re-connection, the producer will publish the content again when connected with the zone 2 router. But as we divided the network into overlapping regions and transmission range of each router is 50 meters, while the overlapping radio signal region is 10 meters. Thus there is very little chance for a producer to disconnect from either zone network.

#### Scenario 3.

**Fig 10:**
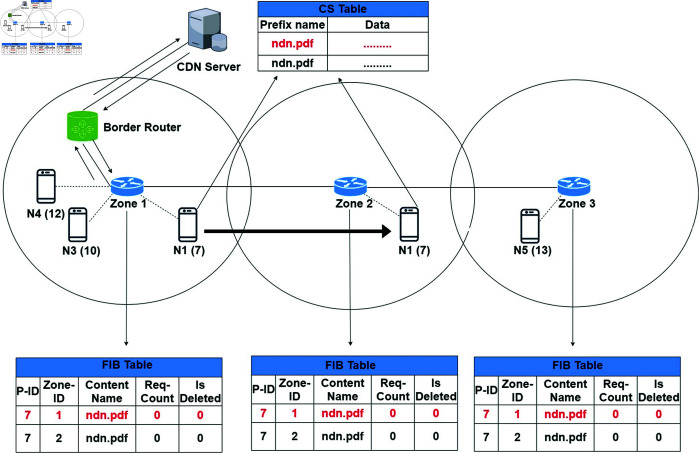
Producer node is disconnected from the zone 1 and connects with zone 2 network.

A producer node "N1" is sending data packets to consumer node "N5" connected in "Z3" shown in Fig 10. During the communication, "N1" performs mobility and receives strong signals from "Z2" and thus connects with "Z2". But in such case, the remaining data packets are forwarded to the "N1" new location at "Z2". "Z2" extracts the consumer’s zone number from the header portion of the data packet and redirects the data packets to the consumer’s zone "Z3". Now the data packet path is changed from "Z2" to "Z3" rather than "Z1" to "Z3". The producer node "N1" also sends the mobility update packet to its "Z2" router to update its new location. The router updates its FIB entry and sends mobility update packets to other zone routers for the producer’s new location. Later, all new content requests for "N1" will be satisfied from "Z2" onwards.

## Performance evaluation

In this section, we describe our proposed ZBP-MM technique with experimental simulation setup and results. To evaluate the proposed technique, it is evaluated and compared against its competitor technique i.e. OPMSS [38].

### Simulation environment

We have implemented the ZBP-MM protocol using the Ubuntu 16.04 operating system with Network Simulator-3 (NS-3) [42] based ndnSIM module [43]. We ran our simulations for three types of scenarios i.e. Sparse, Less Dense, Dense and Very Dense with varying producer and consumer density distributions. In each scenario, the producer and consumer volume density distributions varied across intra-zone and inter-zones. The network zone consisted of 90 nodes acting as producers and consumers with varying speeds. For example, one scenario is a sparse network zone with 20% of producers and consumers moving with nodes moving at speed ranges from 14.4 to 28.8 km/hr (4 to 8 m/s) respectively. The network connectivity type used is WiFi (10 to 50 meters radio range) which varies across zones. The size of the data packet size of 5000 bytes per 10ms delay respectively. A simulation environment summary consisting of the parameters and their values is presented in Table 1.

**Table 1:** Simulation parameters.

Parameters	Values
Simulator	NS-3 based ndnSIM module
No of nodes	90 nodes
Number of zones	3
Simulation area	170 x 250 meters
Producers	Incremental
Consumers %	Sparse 6(20%), Very Dense 9(30%), Dense 12(40%), Very Dense 15(50%)
Network connectivity	WiFi (802.11a)
y Types of scenarios	Sparse, Less Dense, Dense, Very Dense
Mobility speed	4 to 8 m/sec
Movement model	Constant Position Mobility model, Random Walk 2D Mobility Model
Data packet size	5 KB
Interest Dissemination Frequency	2 packets / seconds
Traffic type	Constant Bit Rate
Simulation time	100 seconds
Cache size	10 MB
Content Store (CS) policy	Round Robin
Wired / Wireless link delay	2 and 10 ms
Jitter	0 to 10 ms

### Simulation parameters

**Fig 11:**
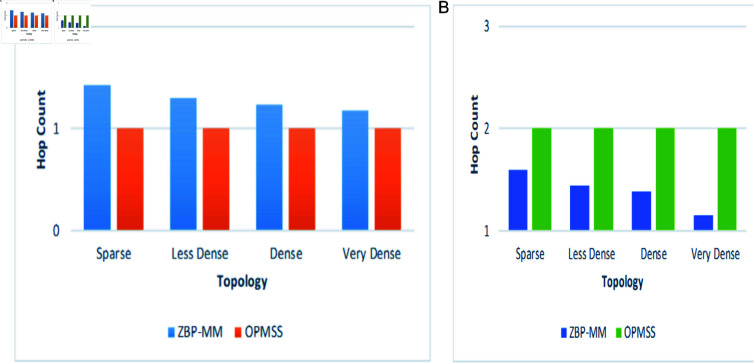
Influence of topological scenarios on hop count in intra-zone and inter-zone.

The simulation environment snapshots of the proposed ZBP-MM technique are illustrated in Fig 11a. The performance parameters which are used to evaluate the performance of the proposed ZBP-MM and comparative OPMSS scheme include Hop Count, Cache Hit Ratio Interest Satisfaction Delay and Handover Delay. We have discussed all those parameters in detail in the following paragraphs (Fig 11).

1. Hop Count: The number of hops the data packets travels from the producer node to the consumer node in the network.
2. Cache Hit Ratio (%): Cache Hit Ratio is to divide the total number of cache hits by the sum of total number of cache hits and cache misses. It measures how many contents are successfully satisfied by the cache. It is represented by the following equation.
  $$CHR=\frac{\sum_{c=1}^{C}N_{c}}{\sum_{s=1}^{S}N_{s}}$$ (1)
  Here *N*_*c*_ is the total number of cache hit count of each requestor node, *N*_*s*_ is the sum of cache hits and cache miss. i.e. total number of data requests.
3. Interest Satisfaction Delay (millisecond): The time interval between first interest packet sent by the consumer and the data packet received by the consumer. It is represented by the following equation.
  $$ISD=T_{I}+T_{I}^{pr}+T_{D}$$ (2)
  Here *T*_*I*_ is the time taken by interest packet to reach the producer, and ${T_{I}}^{pr}$ is the processing time at the producer end and *T*_*D*_ is the time taken by the data packet to reach again to the requestor node.
4. Handover Delay (milliseconds): The time interval between the last received packet from the old PoA at old network until the first received packet from the new PoA at new network. The following equation (3) represents the Total Communication Link Delay during handover.
  TotalCLD=D(MAC−Handover)+D(PN−oCR)+D(oCR−nCR)+D(PN−nCR) (3)
  Here *D*(*MAC*–*Handover*) is the MAC layer link delay during the handover, *D*(*PN*–*oCR*) and *D*(*PN*–*nCR*) are handover delays of producer node from old CR to new CR during the change in PoA, while *D*(*oCR*–*nCR*) is the link delay from old CR to new CR during the handover.

### Results and discussions

**Fig 12:**
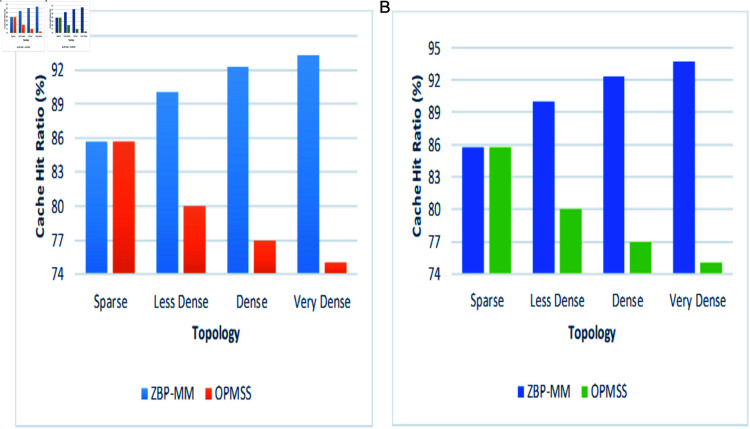
Influence of topological scenarios on cache hit ratio in intra-zone and inter-zone.

The performance of hop count is evaluated under varying topological scenarios and network connectivity in intra-zone (same zone) and inter-zone (adjacent zone) settings. In Fig 12(a), the hop count of ZBP-MM is slightly higher than 1 when compared to OPMSS in the sparse scenario (i.e., low network connectivity). This is because each content router reroutes interest packets and fetches content from the CDN, thereby resulting in a higher hop count than OPMSS. As the network becomes denser, the hop count of ZBP-MM is observed to decrease, approaching a value of 1. This is because each content router finds more producers in its vicinity, thereby increasing the probability of satisfying consumers with the data at shorter hops. In contrast, the hop count of the OPMSS technique remains stable and consistently low at 1, irrespective of network connectivity in either scenario. This stability is attributed to the content zone routers always forwarding interests within the intra-zone to the most recent location of the given producer, thereby maintaining a constant and lower hop count compared to the ZBP-MM technique.

In Fig 12(b), 12(c), the hop count of the ZBP-MM technique under the inter-zone (Adjacent Zone) is recorded as slightly higher than 1 value, this is because the content request packets are fetched from the inter-zone and later content is replicated and later similar content requests are satisfied from same zone, especially in the denser scenario. Moreover, ZBP-MM finds the shortest optimal paths from the number of producers to consumers. Thus lowest hop count is achieved. On the other hand, the hop count of OPMSS remains constant and high at 2 values in the adjacent zone. This is because the content zone routers do not share content information and interface knowledge among themselves in the inter-zones, thus the interest packets are routed towards adjacent zones to satisfy the interest packets (Fig 12).

**Fig 13:**
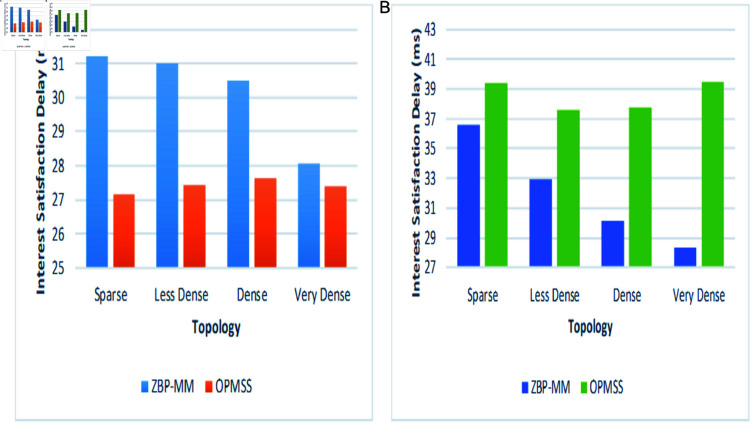
Influence of topological scenarios on cache hit ratio in intra-zone and inter-zone.

Second, we analyzed the performance of the cache hit ratio by varying topological scenarios in intra-zone (Same Zone) and inter-zones (Adjacent Zone). It is observed from the Fig 13(a) ZBP-MM outperforms OPMSS with varying scenarios. However, in sparse scenario there is a cache miss for any consumer requests mainly because the content zone router does not have content information in its FIB table until requests from the CDN-Server replied to the consumer. Now onwards, if a consumer requests the same content then we have a cache hit as the content is available within the zone. If there is a case, where a producer’s data is deleted but information was not updated on the zone router, then cache miss will be observed but according to the ZBP-MM algorithm, similar requests will be satisfied from the other producers resulting in lower cache miss probability. As compared to OPMSS, even with the change in PoA of the producer, the interest packets are satisfied in the intra-zone, if the producer is available. But what if a producer can’t reply to the data, then all the other content requests will have cache misses. The situation becomes worse in denser scenarios if producer data is deleted ultimately, all the requests have cache misses. But in ZBP-MM as the scenario is getting denser, the content has more replicas in the network so the cache miss chances are very low. In the graph, you can see that if accidentally a producer fails to respond, the ZBP-MM hit ratio is not much affected as compared to OPMSS.

By looking at the Fig 13(b) and Fig 13(c), similar inter-zone cache hit ratio, higher cache hit ratio of 93.75 can be observed in ZBP-MM as compared to 73 in OPMSS in intra-zone. This is because, of the possible availability of the same content on two different producers in the different zones. In this case, the data from the nearest producer will be fetched. This will improve the cache hit ratio considerably more than OPMSS (Fig 13).

**Fig 14:**
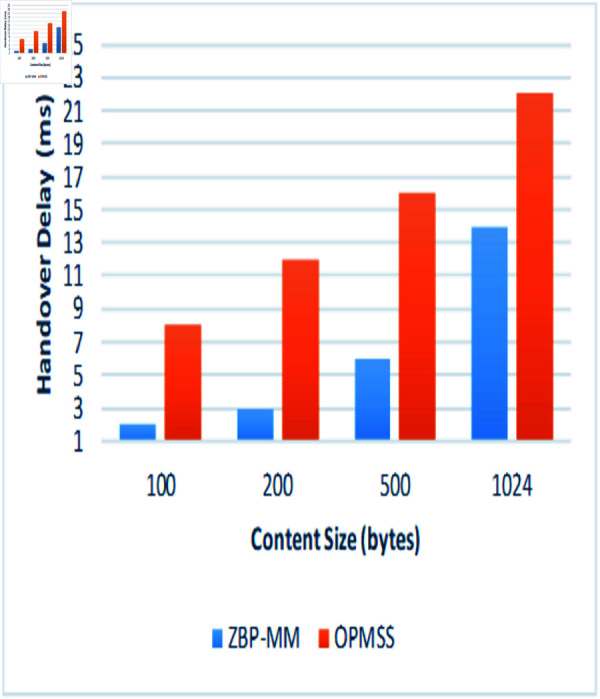
Influence of content size on handover delay in intra-zone and inter-zone.

Third, we measured the performance of Interest Satisfaction Delay by varying topological scenarios in intra-zone (Same Zone) and inter-zones (Adjacent Zone). In Fig 14(a), the Interest Satisfaction Delay of ZBP-MM is recorded higher around 31.2 ms than OPMSS at low network connectivity. This is mainly due to no content availability at the zone, thus higher time taken for first interest packets sent till data packets received due to fetching of content from CDN at the start of the network. But as number of connectivity among nodes increases i.e. from sparse to denser scenario, the interest satisfaction delay declines and recorded 28.07 ms than OPMSS (i.e. 27.4 ms). This is since; consumers can easily find producers at shorter distance with least link failure probability in the zone. On the other hand, OPMSS outperformed ZBP-MM and showed lowest interest satisfaction delay of 27.16 ms in the sparse scenario. This is because, due to producer mobility in intra-zone, the interest packets are satisfied in the same zone rather than fetching data from CDN Server which induces some interest satisfaction delay.

In Fig 14(b), 14(c), the Interest Satisfaction Delay of OPMSS linearly increases with varying scenario and recorded highest value of 39.5 ms and 42.86 ms in very dense scenario. As compared to ZBP-MM, the interest satisfaction delay declined rapidly and recorded the lowest value of 28.33 ms in the same scenario. This is due to the fact that, the number of link failures in the OPMSS network is high due to producer mobility to the inter-zones, thus the content routers are forwarded over longer reliable routes till the producer new PoA so, a higher interest satisfaction delay is achieved. In the ZBP-MM technique, irrespective of link failures with producers, the same content requests are satisfied from other available intra-zone producers as the scenario becomes denser.

Fourth, we examined the performance of handover delay by varying content sizes in intra-zone and inter-zones (Same and Adjacent Zones). By looking at Fig 14, the handover delay linearly increases as content size increases. But ZBP-MM showed the least handover delay of 2 ms at 1024 bytes content size than OPMSS. This is because, in ZBP-MM, when a producer performs mobility and connects new PoA then consumer requests are redirected to a new zone router, to reduce delay in data packet reception at the consumer end. An increasing number of data packets in the content increases the handover delay due to data packet transmission delay. On the contrary, the content zone routers broadcast the mobility interest packet after detecting changes in the PoA of the producer within the zone or outside the zone, the pending interest packets are forwarded to the producer’s new PoA after FIB table update that increases handover delay to 22 ms for 1024 bytes content than ZBP-MM technique.

## Conclusion

One of the primary challenges in named-data networking is the producer mobility problem, which arises due to rapid changes in the producer’s position and routing paths. These changes often result in communication link breakages between consumers and producers, leading to higher packet loss. As a result, consumers flood the network with interest packets in an attempt to locate the content source, increasing network overhead. Addressing this issue requires effective producer mobility management and timely updates to routing paths across zones. Without these measures, networks face increased handover delays and signalling overhead within and across zones or domains. Existing NDN-based producer mobility approaches have struggled to address the timely path update and interest broadcasting challenges in both intra- and inter-zone scenarios, making them unsuitable for resource-constrained NDN-based IoT environments. To address this issue, the proposed ZBP-MM protocol is specifically designed for such environments. It minimizes interest satisfaction delays and handover delays by ensuring up-to-date mobility information is shared across zones or domains. Additionally, a simple path update mechanism is introduced. When a producer changes its location in the network, the zone routers promptly update their FIB tables, even as the producer transitions between Points of Attachment (PoAs) across zones.

## References

[1] Zhang L, Estrin D, Burke J, Jacobson V, Thornton JD, Smetters DK, et al. Named data networking (ndn) project. Relatório Técnico NDN-0001, Xerox Palo Alto Research Center-PARC. 2010;157:158.

[2] Xylomenos G, Ververidis CN, Siris VA, Fotiou N, Tsilopoulos C, Vasilakos X, et al. A survey of information-centric networking research. IEEE Commun Surv Tutorials. 2014;16(2):1024–49. doi: 10.1109/surv.2013.070813.00063

[3] Tariq A, Rehman RA, Kim B-S. Forwarding strategies in NDN-based wireless networks: a survey. IEEE Commun Surv Tutorials. 2020;22(1):68–95. doi: 10.1109/comst.2019.2935795

[4] Zhang M, Luo H, Zhang H. A survey of caching mechanisms in information-centric networking. IEEE Commun Surv Tutorials. 2015;17(3):1473–99. doi: 10.1109/comst.2015.2420097

[5] Zheng J, Simplot-Ryl D, Bisdikian C, Mouftah H. The internet of things [Guest Editorial]. IEEE Commun Mag. 2011;49(11):30–1. doi: 10.1109/mcom.2011.6069706

[6] Al-Fuqaha A, Guizani M, Mohammadi M, Aledhari M, Ayyash M. Internet of Things: a survey on enabling technologies, protocols, and applications. IEEE Commun Surv Tutorials. 2015;17(4):2347–76. doi: 10.1109/comst.2015.2444095

[7] Ericsson L.Cellular IoT evolution for industry digitalization. White Paper; 2019. p. 17.

[8] Amadeo M, Campolo C, Quevedo J, Corujo D, Molinaro A, Iera A, et al. Information-centric networking for the internet of things: challenges and opportunities. IEEE Network. 2016;30(2):92–100. doi: 10.1109/mnet.2016.7437030

[9] Nour B, Ibn-Khedher H, Moungla H, Afifi H, Li F, Sharif K, et al. Internet of Things mobility over information-centric/named-data networking. IEEE Internet Comput. 2020;24(1):14–24. doi: 10.1109/mic.2019.2963187

[10] Fang C, Yao H, Wang Z, Wu W, Jin X, Yu FR. A survey of mobile information-centric networking: research issues and challenges. IEEE Commun Surv Tutorials. 2018;20(3):2353–71. doi: 10.1109/comst.2018.2809670

[11] Sheng Z, Yang S, Yu Y, Vasilakos A, Mccann J, Leung K. A survey on the ietf protocol suite for the internet of things: standards, challenges, and opportunities. IEEE Wirel Commun. 2013;20(6):91–8. doi: 10.1109/mwc.2013.6704479

[12] Aboodi A, Wan T-C, Sodhy G-C. Survey on the Incorporation of NDN/CCN in IoT. IEEE Access. 2019;7:71827–58. doi: 10.1109/access.2019.2919534

[13] Mwangi XK. Scalable mobility support in future internet architectures. Massachusetts Institute of Technology; 2018.

[14] Ahlgren B, Dannewitz C, Imbrenda C, Kutscher D, Ohlman B. A survey of information-centric networking. IEEE Commun Mag. 2012;50(7):26–36. doi: 10.1109/mcom.2012.6231276

[15] Alajlan M, Belghith A. Supporting seamless mobility for real-time applications in named data networking. Procedia Comput Sci. 2017;110:62–9. doi: 10.1016/j.procs.2017.06.117

[16] Zafar WUI, Rehman MAU, Jabeen F, Ullah R, Abbas G, Khan A. Decentralized receiver-based link stability-aware forwarding scheme for NDN-based VANETs. Computer Networks. 2023;236:109996. doi: 10.1016/j.comnet.2023.109996

[17] Rui L, Dai S, Gao Z, Qiu X, Chen X. Double-lead content search and producer location prediction scheme for producer mobility in named data networking. Comput J. 2022;66(11):2825–43. doi: 10.1093/comjnl/bxac125

[18] Fayyaz S, Atif Ur Rehman M, Khalid W, Kim B-S. SHM-NDN: a seamless hybrid mobility management scheme for named data mobile ad hoc networks. Internet of Things. 2023;24:100943. doi: 10.1016/j.iot.2023.100943

[19] Kar P, Chen R, Qian Y. An efficient producer mobility management technique for real-time communication in NDN-based Remote Health Monitoring systems. Smart Health. 2022;26:100309. doi: 10.1016/j.smhl.2022.100309

[20] Hernandez D, Luis M, Sargento S. Consumer mobility awareness in named data networks. IEEE Access. 2022;10:18156–68. doi: 10.1109/access.2022.3150010

[21] Prates AA, Bastos IV, Moraes IM. GeoZone: an interest-packet forwarding mechanism based on dissemination zone for content-centric vehicular networks. Comput Electric Eng. 2019;73:155–66. doi: 10.1016/j.compeleceng.2018.11.015

[22] Ahmed R, Boutaba R. Design considerations for managing wide area software defined networks. IEEE Commun Mag. 2014;52(7):116–23. doi: 10.1109/mcom.2014.6852092

[23] Rao Y, Gao D, Luo H. NLBA: a novel provider mobility support approach in mobile NDN environment. In: 2014 IEEE 11th Consumer Communications and Networking Conference (CCNC). 2014. p. 188–93. 10.1109/ccnc.2014.6866569

[24] Tang J, Zhou H, Liu Y, Zhang H, Gao D. A source mobility management scheme in content-centric networking. In: 2014 IEEE 11th Consumer Communications and Networking Conference (CCNC). 2014. p. 176–81. doi: 10.1109/ccnc.2014.6866567

[25] Do T, Kim Y. Optimal provider mobility in large-scale named-data networking. KSII Trans Internet Inf Syst. 2015;9(10):4054–71.

[26] Zheng Y, Piao X, Lei K. Anchor-chain: a seamless producer mobility support scheme in NDN. In: 2017 IEEE 85th Vehicular Technology Conference (VTC Spring). 2017. p. 1–6. doi: 10.1109/vtcspring.2017.8108547

[27] Azgin A, Ravindran R, Wang G. On-demand mobility support with anchor chains in information centric networks. In: 2017 IEEE International Conference on Communications (ICC). 2017. p. 1–7. doi: 10.1109/icc.2017.7997210

[28] Ge J, Wang S, Wu Y, Tang H, E Y. Performance improvement for source mobility in named data networking based on global–local FIB updates. Peer-to-Peer Netw Appl. 2015;9(4):670–80. doi: 10.1007/s12083-015-0353-z

[29] Rui L, Yang S, Huang H. A producer mobility support scheme for real-time multimedia delivery in named data networking. Multimed Tools Appl. 2018;77(4):4811–26. doi: 10.1007/s11042-017-5601-1

[30] Zhang Y, Xia Z, Mastorakis S, Zhang L. Kite: producer mobility support in named data networking. In: Proceedings of the 5th ACM Conference on Information-Centric Networking; 2018. p. 125–36.

[31] Auge J, Carofiglio G, Grassi G, Muscariello L, Pau G, Zeng X. MAP-Me: managing anchor-less producer mobility in content-centric networks. IEEE Trans Netw Serv Manage. 2018;15(2):596–610. doi: 10.1109/tnsm.2018.2796720

[32] Hussaini M, Nor SA, Ahmad A. Optimal broadcast strategy-based producer mobility support scheme for Named Data Networking. 2019.

[33] Ali I, Lim H. Anchor-less producer mobility management in named data networking for real-time multimedia. Mobile Inf Syst. 2019;2019:1–12. doi: 10.1155/2019/3531567

[34] Farahat H, Hassanein HS. Proactive caching for Producer mobility management in Named Data Networks. In: 2017 13th International Wireless Communications and Mobile Computing Conference (IWCMC). 2017. p. 171–6. doi: 10.1109/iwcmc.2017.7986281

[35] Araújo FRC, de Sousa AM, Sampaio LN. SCaN-Mob: an opportunistic caching strategy to support producer mobility in named data wireless networking. Comput Netw. 2019;156:62–74. doi: 10.1016/j.comnet.2019.04.008

[36] Meddeb M, Dhraief A, Belghith A, Monteil T, Drira K, Gannouni S. AFIRM: adaptive forwarding based link recovery for mobility support in NDN/IoT networks. Future Gen Comput Syst. 2018;87:351–63. doi: 10.1016/j.future.2018.04.087

[37] Hussaini M, Naeem MA, Kim B-S, Maijama’a IS. Efficient producer mobility management model in information-centric networking. IEEE Access. 2019;7:42032–51. doi: 10.1109/access.2019.2907653

[38] Hussaini M, Naeem MA, Kim B-S. OPMSS: optimal producer mobility support solution for named data networking. Appl Sci. 2021;11(9):4064. doi: 10.3390/app11094064

[39] Gohar M, Khan N, Ahmad A, Najam-Ul-Islam M, Sarwar S, Koh S-J. Cluster-based device mobility management in named data networking for vehicular networks. Mobile Inf Syst. 2018;2018:1–7. doi: 10.1155/2018/1710591

[40] Korla S, Chilukuri S. T-Move: a light-weight protocol for improved QoS in content-centric networks with producer mobility. Future Internet. 2019;11(2):28. doi: 10.3390/fi11020028

[41] Serhane O, Yahyaoui K, Nour B, Moungla H. A label-based producer mobility support in 5G-enabled ICN networks. In: 2020 International Wireless Communications and Mobile Computing (IWCMC). 2020. p. 2094–9. doi: 10.1109/iwcmc48107.2020.9148578

